# Research Brief: Self-Reports of a Constellation of Persistent Antiandrogenic, Estrogenic, Physical, and Psychological Effects of Finasteride Usage Among Men

**DOI:** 10.1177/1557988317750989

**Published:** 2018-01-10

**Authors:** Alicia A. Walf, Shan Kaurejo, Cheryl A. Frye

**Affiliations:** 1 Departments of Psychology, The University at Albany-SUNY, Albany, NY, USA; 2 Department of Cognitive Science, Rensselaer, Troy, NY, USA; 3 The Centers for Life Sciences, The University at Albany-SUNY, Albany, NY, USA; 4 Neuroscience Research, The University at Albany-SUNY, Albany, NY, USA; 5 Biological Sciences, The University at Albany-SUNY, Albany, NY, USA

**Keywords:** androgens, testosterone, sexual dysfunction, depression, cognitive performance

## Abstract

Our research objective is to understand more, through subjective, self-reports on discussion boards/forums, persons’ experiences associated with the use of drugs that alter androgen metabolism, such as finasteride. Finasteride is an orally active, specific inhibitor of 5α-reductase, which is localized to many androgen-dependent tissues. Finasteride inhibits the conversion of testosterone (T) to dihydrotestosterone (DHT), and is commonly used to treat benign prostatic hypertrophy (BPH) and male pattern baldness (MPB), both disorders associated with elevated DHT levels and 5α-reductase activity in the prostate and hair follicles, respectively. It is now acknowledged that long-term use and discontinuation of finasteride has adverse effects (AEs); however, these claims have not been well documented. In this study, discussion board posts (forums) were analyzed as self-reports of what finasteride users indicate is problematic for them. Reports were categorized by the age of subjects as well as the types of AEs described: antiandrogenic, estrogenic, central, and nonspecific/severe. A total of 244 cases were recorded and analyzed on the discussion forum on propeciahelp.com. Among these, 74 (32%) cases reported antiandrogenic affects, 43 (19%) reported estrogenic effects, 70 (30%) reported central effects, 11 (5%) reported nonspecific/severe AEs, and 31 (14%) reported AEs in all categories. The categorization of AEs may prompt further investigation into the pathophysiology of post-finasteride syndrome (PFS). Also, subjective reports may engender greater understanding of the perceived lasting AEs of finasteride.

## Introduction: Current Research Findings Regarding Persistent Finasteride Effects

Steroid hormones, including androgens, such as testosterone, exert many effects, which may be due in part to actions of their metabolites. Androgens’ actions involve its metabolism by 5α-reductase enzymes, which convert the body’s main androgen, testosterone, to dihydrotestosterone (DHT; Kohtz & Frye, 2012). Finasteride is an orally active, specific inhibitor of 5α-reductase, which is localized to many androgen-dependent tissues. Benign prostatic hypertrophy (BPH) and male pattern baldness (MPB; aka androgenic alopecia) are androgen-dependent disorders, associated with high levels of DHT and increased 5α-reductase activities in prostate and hair follicles, respectively. Both BPH and MPB respond favorably to treatment with finasteride, with reductions in DHT and suppression of 5α-reductase (Traish, Melcangi, Bortolato, Garcia-Segura, & Zitzmann, 2015a). There can also be considerable adverse effects (AEs) of finasteride. These may be due to its nonspecific effects on 5α-reductase expression in reproductive organs, brain, and/or peripheral organs (e.g., cardiovasculature). As well as altering 5α-reductase to reduce DHT, other enzymes such as aromatase and its products, respond to finasteride therapy (neurosteroids; Caruso et al., 2015; Melcangi et al., 2013). Castro-Magana and colleagues documented an inverse pattern between P450 aromatase and 5α-reductase activities. It is hypothesized that DHT inhibits aromatase and that finasteride is likely responsible for the moderate increase in estrogens found in some patients on finasteride therapy (Castro-Magana et al. 1996). Moreover, some AEs of finasteride may continue despite discontinuation of finasteride. Post-finasteride syndrome (PFS) is a condition associated with altered steroid levels and these varied AEs that persist after treatment cessation (Ganzer, Jacobs, & Iqbal, 2014; Irwig, 2012a, 2012b, 2014a, 2014b; Irwig & Kolukula, 2011; Traish et al., 2015a, Traish, Haider, Doros, & Haider, 2015b). These symptoms can range from mild to severe and can be physical or psychological, including changes in sexual function, sleep/energy, growth and/or shrinkage of tissues, in sensory processes, and/or higher cognitive functions, including mood. Simply, there is general functional decline following finasteride usage among some men.

One way to understand symptoms following finasteride treatment and its discontinuation is to assess subjective reports. Many individuals blog about their experiences on propeciahelp.com (and other websites). A recently published study used a survey method to characterize this constellation of symptoms in men that used finasteride to treat MPB, and stopped taking the drug for at least 3 months prior to assessment (of symptoms) (Ganzer et al., 2015). The Internet-based survey that was emailed to participants (*N* = 131, mean age of 24, range 21–62) who reported experiencing symptoms of AEs of finasteride, targeted six domains: (a) physical symptoms, (b) sexual libido, (c) ejaculatory disorders, (d) disorders of the penis and testes, (e) cognitive symptoms, and (f) psychological symptoms. The majority of the sample reported several symptoms across domains that they believed was associated with their past finasteride use. A striking physical symptom, reported by 91 individuals (or 70% of the sample) was gynecomastia (irreversible enlargement of the breasts). Other physical symptoms reported by nearly 70% of the sample was lethargy/fatigue and dry, thinning skin. Changes in muscle strength and changes in metabolism/fat deposition were reported in the majority of subjects (56% and 54%, respectively). As for three domains associated with sexual function (sexual libido, ejaculatory disorders, and disorders of the penis and testes), nearly all subjects reported negative changes in libido (93%), loss of morning erections (89%), erectile dysfunction (83%), and negative changes in ejaculation (82%). Penile and scrotal shrinkage and loss of sensation were reported by approximately 80% of subjects. Anhedonia (loss of pleasure) in sex and in general was reported in about 70% of subjects. The most commonly reported cognitive symptoms were: slowed thought processes (74%), mental cloudiness/“brain fog” (75%), and attentional difficulties (74%). Confusion and severe memory recall impairment were also reported in more than 50% of subjects. As for psychological symptoms, elevated anxiety and depressed affect were reported in many subjects, 73% and 74% of the sample, respectively. Sleep disturbance was reported among a significant number of subjects (58%). As well, suicide ideation or feeling hopelessness regarding coping with the AEs of finasteride on a daily basis was reported among 82, or 68%, of the respondents. Simply, the results from this study demonstrated that there were persistent AEs in each of the six domains assessed and, thereby, showing support for the presence of both physical and psychological changes following finasteride use. This is significant because the intended use of finasteride is for physical symptoms associated with androgens, that is, hair loss and/or prostate hyperplasia. Albeit, laboratories studying the role of brain 5α-reductase for androgen action, and finasteride as a tool to alter metabolism or neurosteroid synthesis in the brain, have been using finasteride as a pharmacological tool in animal models for decades (e.g., Frau et al., 2013; Ford, Nickel, & Finn, 2005; Frye, Scalise, & Bayon, 1998; Frye & Walf, 2002; Serra, Sanna, Mostallino, & Biggio, 2007).

In the present report, our aim was to categorize the nature of these physical and psychological effects. This report briefly details a similar pattern of responses when using another approach to describe the types and incidence of symptoms in the same online forum, propeciahelp.com. Looking at these subjective reports, which were provided anonymously and spontaneously (i.e., not in response to direct questioning in a survey or from a clinician) may provide important information for the greater understanding of the perceived AEs of finasteride. As such, 224 discussions on propeciahelp.com forum were collected from a discrete time period and were analyzed and categorized based on self-reports into antiandrogenic (demasculinizing), estrogenic (feminizing), central/brain effects, and nonspecific/severe AEs (i.e., those not falling into these other three categories).

## Methods: Design and Data Collection

This project was reviewed and approved by the Institutional Review Board at University at Albany, SUNY (Albany, NY). Information from discussion forums, sampled from Fall 2012, publically available on the Web, about self-reports of effects of finasteride were recorded and analyzed. This discrete time period was utilized because it coincided with the months when an undergraduate research assistant was being mentored on this project.

In general, reports of AEs were categorized as antiandrogenic, estrogenic, central, and nonspecific/severe AEs. A description of these and specific examples are provided in Table 1. Demasculinizing (antiandrogenic) effects occur due to androgen (specifically DHT) deprivation of the sexual organs. Feminizing (estrogenic) effects occur due to increased estrogen activity. Central/brain effects occur due to inhibition of 5α-reductase and other enzymes/products, reduced levels of neurosteroids or cerebral thromboembolic events. Nonspecific/severe AEs are side effects that were vague but still serious; this category was utilized to note other AEs that could not be considered clearly antiandrogenic, estrogenic, or central/brain effects. These categorical definitions provided the criteria to analyze and group the self-reports systematically. The reports were assessed and categorized by two individuals (the research assistant, AD, and mentor, CAF). When it was disclosed, the age of the blogger was reported.

**Table 1.** Types of AEs: The Categories of Side-Effects Used Below Are Congruous With the Adverse Event Reporting System for the FDA.

**Antiandrogenic effects** The adverse events in this group fall into five subgroups and include one or more of the following:	(a) Genital function: (i) erectile dysfunction, impotence, anorgasmia, abnormal orgasm or male orgasm disorder, genital disorder or sexual dysfunction; (ii) ejaculation disorder or ejaculation failure; (iii) shrinkage or reduction in size of penis, penis disorder or Peyronie’s disease; sensory loss, hypoaesthesia or paresthesia of genitals; and (iv) painful erection; penile or genital pain; and penile abscess (b) Testicular function and infertility: (i) infertility; abnormal semen analysis, viscosity or volume, or semen discolouration; abnormal spermatogenesis, azospermia, hematospermia, or abnormal sperm count, motility or morphology; (ii) testicular pain, testicular disorder, testicular tortion, testicular atrophy, testicular swelling, orchitis, or scrotal pain; and (iii) seminoma or testicular neoplasm or cancer (c). Accessory sexual or genitourinary organ function: (i) epididymal cyst, epididymitis, or seminal vesiculitis; (ii) prostate cancer or bladder cancer; and (iii) prostatitis, prostatic pain, or prostatic or anorectal disorder; perineal or pelvic pain; and urinary incontinence or micturition disorder (d) Psychosexual function: decreased libido and psychosexual disorder (e) Hormonal function: (i) decreased blood testosterone levels; and (ii) abnormal blood FSH or LH levels
**Estrogenic effects** The AEs in this group include one or more of the following in the absence of any reported antiandrogenic effects:	(a) Male breast cancer (b) Breast neoplasm or breast mass (c) Gynecomastia, breast enlargement, breast pain, or breast disorder (d) Increased blood estrogen levels
**Central effects** The AEs in this group include one of more of the following in the absence of any reported antiandrogenic or estrogenic effects:	(a) Convulsion, grand-mal convulsion, seizures or petite mal epilepsy; and loss of consciousness or syncope (b) Cerebral thromboembolic events (e.g., cerebrovascular accident, cerebral infarction or transient ischemic attack; and cerebral artery thrombosis, cerebral venous thrombosis or intracranial venous sinus thrombosis); and hydrocephalus (c). Depression, apathy, crying, or anhedonia; completed suicide, suicide attempt, or suicidal ideation; committed homicide or homicidal ideation; catatonia or malignant neuroleptic disorder; and affective or emotional disorder (d) Anxiety, nervousness, restlessness, irritability, and confusion (e) Confusional state or agitation; suffocation feeling or claustrophobia; aggression, anger, hyperactivity, mania, or bipolar disorder; panic disorder or agoraphobia; and obsessive–compulsive personality disorder or obsessive thoughts (f) Psychotic disorder or acute psychosis; and paranoia, hallucinations, or auditory or persecutory delusions (g) Personality change and mental impairment or disorder (h) Insomnia, hypersomnia, or sleep disorder (i) Attention deficit or disturbance; attention deficit hyperactivity
**Nonspecific and severe AEs** The AEs in this group include one of more of the following in the absence of any reported antiandrogenic, estrogenic or central effects:	(a) Serious cardiac AEs (e.g., myocardial infarction, coronary artery ischemia, atrial fibrillation, bundle branch block, etc.) (b) Potential life-threatening AEs related to cardiac arrhythmias, vascular disease, or abnormal coagulation (e.g., cardiac arrest, ventricular tachycardia, ventricular fibrillation, pulmonary embolism, arterial thrombosis, venous thrombosis of major vessels, acute renal failure or infarction, idiopathic/thrombotic thrombocytopenic purpura, systemic lupus erythematosus, etc.) (c). Malignant neoplasm of unspecified origin (d) Hypotonia, muscular weakness, muscle spasms or myalgia (e). Paresthesia or hypoesthesia

*Note.* Subject self-reports on propeciahelp.com were collected, categorized, and recorded based on the following types of effects. AEs = adverse effects; FDA = food and drug administration; FSH = follicle-stimulating hormone; LH = luteinizing hormone.

## Findings

The majority of posts that were analyzed did not indicate the age of the individual who was self-reporting (141, or 63% of 244 total cases reviewed). In the cases where an age was indicated, few cases of the samples were from the age groups 10–19 or 40+ years of age (*n* = 4 or 6, respectively). Of those cases in which age was reported, 49 (59% of 83 cases) were in the 20–29 age group and 24 (29%) were in the 30–39 age group. Of the 244, there were 20 posts that did not report their age or AEs of finasteride (8% of the sample); the vast majority of posts indicated AEs (Table 2).

**Table 2.** Summary of Cases Reviewed: A Breakdown of the 244 Cases That Were Analyzed.

Number of cases reviewed (*N* = 244)	Self-reported age or none	Reported antiandrogenic effects	Reported estrogenic effects	Reported central effects	Reported nonspecific AEs and selected severe AE	Reported AEs in all categories
**141** (63%)	Not reported	56	25	35	5	20
**4** (2%)	10–19	2	0	2	0	0
**49** (22%)	20–29	7	14	19	2	7
**24** (11%)	30–39	5	3	8	4	4
**6** (2.5%)	40+	4	1	1	0	0
**20**	Not reported	Not reported				
**224**	10–40+	74 (32%)	43 (19%)	65 (30%)	11 (5%)	31 (14%)

*Note.* When indicated, the number of cases within age groups is described. The number of cases reporting antiandrogenic, estrogenic, central, and nonspecific AEs, or cases where all types of AEs were reported, is indicated; in the first column and in the last row, the percentage of the group is indicated in parentheses. AEs = adverse effects.

A breakdown of the number of 244 cases reported one of the four categories (antiandrogenic, estrogenic, central/brain, nonspecific/severe) is indicated in Table 2. A total of 74 (32%) cases reported antiandrogenic effects, 43 (19%) reported estrogenic effects, 70 (30%) reported central effects, 11 (5%) reported nonspecific AEs. A subset of individuals reported AEs that fell into all of the categories (31% or 14%).

## Discussion: Recommendations For Further Study and Categorization of Persistent Finasteride Effects

The present report describes a constellation of symptoms following use and discontinuation of finasteride. The antiandrogenic AEs included genital dysfunction, testicular function and infertility, accessory sexual or genitourinary organ dysfunction, psychosexual function, and hormonal function. The estrogenic AEs included breast cancer, breast neoplasm or breast mass, gynecomastia, breast pain, and increased blood estrogen levels. The central effects consisted of depression, anxiety, confusion/“brain fog,” and sleep and attentional problems. The nonspecific/severe AEs consist of muscle twitching, lower back pain, weight gain, fatigue, numbness in the anal region, muscle spasms, excessive sweating, bleeding gums, tinnitus, hot flashes, irregular stool, scoliosis, and discoloration of the urine. In some individuals, there were reports of AEs from all these categories. Importantly, this study corroborates another using self-reports as discussed in the Introduction (see (Ganzer et al., 2015) as well as other methods to begin to characterize symptoms after finasteride is discontinued as described in the next paragraphs.

Other approaches have been utilized to assess these AEs among patients, showing converging evidence about the nature of the AEs of finasteride use that can persist after discontinuation. Antiandrogenic effects, generally considered sexual side effects, can include an overall decrease in libido/sexual drive, erectile and ejaculatory dysfunctions, and negative physical and sensory changes to the penis and scrotum and have been reported in previous studies (Carbone & Hodges, 2003; McClellan & Markham, 1999; Irwig, 2012b, 2014; Irwig & Kolukula, 2011; Mella, Perret, Manzotti, Catalano, & Guyatt, 2010; Traish, Hassani, Guay, Zitzmann, & Hansen, 2011; Traish et al., 2015a, 2015b). A prospective study of otherwise healthy men, 40 years old and younger (*N* = 54) who reported persistent AEs of finasteride, showed that sexual symptoms endured in many respondents at assessments at 3, 9, and 16 months following drug discontinuation (Irwig, 2012b). The estrogenic effects, including gynecomastia, are not new and were reported about 20 years ago by Kaufman and colleagues (Kaufman et al., 1998) as well as the U.S. Food and Drug Administration (FDA). Although a benign condition, gynecomastia may cause substantial anxiety and discomfort; however, it is a commonly overlooked AE by practicing clinicians. This is one example where a physical AE may contribute to a psychological AE. Although the aim of this study was to assess the occurrence of both physical and psychological symptoms, other work needs to be done to determine the relationships between these.

The central effects associated with the use of finasteride, include cognitive and affective/psychological changes. Yet, these effects are not well studied compared to AEs on sexual function, partly because of the implicit, but incorrect, assumption that finasteride does not target brain 5α-reductase. As in the present report, the self-reported central symptoms can include mental cloudiness, difficulties with attention/focus, and memory problems (Ganzer et al., 2015). There is also evidence of finasteride altering other complex cognitive behaviors, such as motivation and reward. This has been observed in case studies and clinical reports showing that finasteride can reduce symptoms across different neuropsychiatric or neurodegenerative disorders (reviewed in Traish et al., 2015; Paba et al., 2011). For example, finasteride reduced, tics, a major symptom, in Tourette Syndrome (Muroni, Paba, Puligheddu, Marrosu, & Bortolato, 2011). In another case study, problem gambling (as a potential adverse effect of treatment for Parkinson’s disease with dopaminergic agents) was reduced with finasteride administration (Bortolato et al., 2012). These studies demonstrate direct evidence to suggest that finasteride has effects on 5α-reductase in the brain.

There is also ample evidence of adverse psychological or mood effects of finasteride following its discontinuation. In the recently published pilot study using a survey method to characterize psychological (and other) symptoms in men who used finasteride to treat MPB, the cohort reported high rates of psychological symptoms (Ganzer et al., 2015); these effects are much like what we report here using a different approach. Moreover, a recent report has corroborated and extended these results to assess specifically mood symptoms (Ganzer & Jacobs, 2016). Further, in a clinical study, significantly increased depression symptoms and suicidality were reported by three-quarters of the subject group that had discontinued finasteride, compared to about 10% of the control group (Irwig, 2012a). Among patients that have discontinued finasteride treatment, there was depression, mood disturbances, and changes in steroid levels in plasma and cerebrospinal fluid (Melcangi et al., 2013). Another study evaluated the hypothalamic-pituitary-gonadal (HPG) axis, irreversible suppression of SRD5A1 (5α-reductase gene), off-target suppression of androgen receptor (AR) action, effects on brain regions that regulate sexual function and mood, cognitive function, and sexual function in symptomatic finasteride users, asymptomatic finasteride users, and a control group (healthy men under 50 years of age; Basaria et al. 2016). Overall, Basaria et al. found no hormonal or genetic correlate as the causative factor for PFS. However, they observed subjective differences in personality/mood, sexual function, and cognitive function. Importantly, neural activity as determined by functional magnetic resonance imaging blood oxygen level-dependent (fMRI BOLD) activity, showed abnormal neural activity in regions associated with sexual function and major depression only in symptomatic finasteride users. Based on the comprehensive assessment by Basaria et al., it is likely that PFS may be due to a persistent rewiring of neural circuitry that may or may not have a hormonal correlate. Thus, these studies in people show that there are physical and central effects of finasteride that can be persistent, and the hormonal correlates have been hinted at and need further investigation.

In summary, these studies show that there can be effects of finasteride for both physical and psychological indices. Yet, there are potential limitations of this study that cannot be ignored. There may have been selection and recall biases from recruiting subjects who had sought treatment for symptoms from a website; it is unknown whether comments are only in response to others’ comments on the forum. Also, the self-reports assessed here were specifically used to address questions of spontaneous subjective reporting by not using typical laboratory or clinical methods (which may have their own biases to account for) to study the symptoms of the men with PFS. The accuracy of self-reports must be considered mainly because AEs could be due to other reasons that are not PFS and without clinical assessments individuals may misdiagnose/describe inaccurately AEs. Additionally, we are not able to directly link these effects to hormone levels (e.g., it is not known if estrogenic effects are due to high estradiol levels or changes in the ratio of androgens to estrogens). It would be of use to extend this work in future studies that could use validated instruments such as those available for sexual function, depression, anxiety, general overall health, and so on, and hormonal measurements (and which have been reported on elsewhere). Recent research has turned to study the long-term effects of finasteride, or how these occur post-discontinuation. Part of this may be due to the inadequacy of clinical trials (e.g., as reviewed by Mella et al., 2010). Indeed, despite this evidence, there is little mention regarding the potential AEs in the Propecia product insert. However, it should be noted that these findings have led to prompting the FDA to mandate that the labeling of Propecia to include information about potential risks of depression as well as sexual function problems and high-grade prostate cancer (FDA, 2010; Traish et al., 2015a). These studies also provide support for the notion that some men respond poorly to levels of endogenous circulating androgens (e.g., those with MPB and BPH) and/or their manipulation with finasteride administration (e.g., those with PFS symptoms). Additional considerations that may underlie individual differences in responses to finasteride are subject variables, such as the degree of brain lateralization and cognitive style (reviewed in Motofei et al., 2017, 2016a, 2016b). A proxy for brain lateralization, which can reflect cognitive style, is handedness. Although the present study did not account for this, another study demonstrated that short-term finasteride treatment for male pattern baldness had opposite effects on changes in sexual functioning in right- and left-handed subjects (Motofei et al., 2013). Among subjects who reported differences in sexual functioning scores after treatment compared to before treatment, right-handed individuals reported worsening of sexual function, whereas left-handed individuals reported improvements (Motofei et al., 2013). Future work should continue to assess the varied AEs associated with altering androgen metabolism or action, such as these effects described herein on finasteride, as well as other pharmacotherapies.
